# Daptomycin Therapy for Osteomyelitis: A Retrospective Study

**DOI:** 10.1186/1471-2334-12-133

**Published:** 2012-06-12

**Authors:** Jason C Gallagher, Jennifer A Huntington, Darren Culshaw, Scott A McConnell, Minjung Yoon, Elie Berbari

**Affiliations:** 1 School of Pharmacy, Temple University, 3307 N Broad St, Philadelphia, PA 19140, USA; 2 Cubist Pharmaceuticals, Inc, 65 Hayden Ave, Lexington, MA 02421, USA; 3 Department of Medicine, Division of Infectious Diseases, Mayo Clinic College of Medicine, 200 1st St, SW, Rochester, MN 55905, USA

## Abstract

**Background:**

Daptomycin is a rapidly bactericidal agent with broad coverage against Gram-positive organisms, including *Staphylococcus aureus,* the most frequent cause of osteomyelitis. The objective of this study was to describe the clinical outcome of patients with non-hardware associated osteomyelitis, and the safety profile of daptomycin in the treatment of these infections.

**Methods:**

All patients with osteomyelitis, excluding concurrent orthopedic foreign body infections, treated with daptomycin and identified between 2007–2008 in a retrospective, multicenter, observational registry, were included. Investigators assessed patient outcome (cured, improved, failed, non-evaluable) at the end of daptomycin therapy. Patients with a successful outcome at the end of daptomycin therapy were reassessed in 2009. All patients were included in the safety analysis; evaluable patients were included in the efficacy analysis. Data was assessed using descriptive statistics. A Kaplan Meier analysis was used to assess time to clinical failure.

**Results:**

Two-hundred and nine osteomyelitis patients successfully completed daptomycin therapy in 2007–2008, 71 of which (34%) had a follow-up visit in 2009 and had an evaluable clinical outcome. The median (min, max) daptomycin dose and duration were 6 mg/kg (4, 10) and 42 days (1, 88), respectively. Of the 52 patients with a documented pathogen, *S. aureus* was the most common (42%); primarily methicillin-resistant *S. aureus*. All patients were included in the safety analysis; evaluable patients were included in the efficacy analysis. Clinical resolution was reported in 94% (CI - 86.2%, 98.44%) of patients. A Kaplan Meier analysis of time to clinical failure showed that approximately 85% (CI – 64%, 95%) of patients had a continued successful outcome at the time of re-evaluation. Eighteen patients (25%) in the safety population experienced an adverse event; 13 patients (18%) had an adverse event that was possibly-related to daptomycin treatment.

**Conclusions:**

Daptomycin appears to be an effective therapeutic choice with an acceptable safety profile in the management of osteomyelitis that does not involve hardware.

## Background

Osteomyelitis continues to represent a difficult therapeutic challenge. Management of osteomyelitis typically includes a prolonged course of antimicrobial therapy in addition to surgical intervention. *Staphylococcus aureus* is the most frequent cause of osteomyelitis, accounting for >50% of cases [1]. Recent data suggest that methicillin-resistant *S. aureus* (MRSA) is becoming less susceptible to vancomycin, and has been associated with clinical failures when vancomycin is utilized [2-5]. Data from a large cohort of outpatient treatment for *S. aureus* osteomyelitis showed that vancomycin was associated with a 2.5 times higher relative risk of recurrence compared to β-lactams [6]. Therefore, alternatives to vancomycin are necessary.

Daptomycin is rapidly bactericidal against Gram-positive organisms, including methicillin-susceptible *S. aureus* (MSSA), MRSA and coagulase-negative staphylococci. The bactericidal activity of daptomycin is concentration-dependent in vitro and since it is not dependent on cell growth, it maintains activity against bacteria in biofilm or stationary growth phase [7-9]. Daptomycin is not FDA-approved for bone and joint infections [10]; however, animal and clinical data indicate daptomycin has activity. A rabbit experimental model of osteomyelitis showed infection clearance of 90%, 67%, 33% and 13% for daptomycin 25 mg/kg, 15 mg/kg, vancomycin, and untreated controls, respectively. Daptomycin doses of 25 and 15 mg/kg in the rabbit correspond to equivalent human exposures from 8 and 6 mg/kg [11]. Clinical outcomes for osteomyelitis patients from a retrospective, observational, non-comparative registry showed that doses > 4 mg/kg were more effective than doses ≤ 4 mg/kg (88% vs 65%, *P* = 0.013) [12].

The aims of this study were to describe the clinical outcome of daptomycin-treated patients with non-hardware-associated osteomyelitis, as well as describe the safety profile of daptomycin using data collected in a multicenter retrospective registry.

## Methods

Cubicin® Outcomes Registry and Experience (CORE®) is a multicenter, retrospective, observational database designed to collect the demographics, clinical outcome, and safety of patients treated with daptomycin. The methods used to collect data have been previously published [13,14]. Investigators entered sequentially treated patients who received daptomycin at their institution. Patients were not excluded for any underlying disease or clinical presentation. Data for this study came from 54 separate institutions in the United States between January 2007 and December 2008. The study was approved by the investigational review board of the study centres.

All patients with osteomyelitis, excluding concurrent orthopedic foreign body infections, who successfully finished therapy with daptomycin were identified. To determine the persistence of clinical success, patients with a successful outcome at the end of daptomycin therapy in 2007 through 2008 were reassessed in 2009 for any contact with their physician or institution related to their osteomyelitis treatment. Patients that had a follow-up visit in 2009 were considered eligible for inclusion in this study.

Efficacy of daptomycin at the end of therapy and at follow-up was determined on the basis of the clinical response of the patient as determined by the investigator using a standardized definition. These definitions were designed to be applicable to a wide range of infectious processes and all qualifiers may not have been used for the osteomyelitis patients in this study. Clinical response was defined as resolved (any of the following: clinical signs and symptoms are resolved and/or no additional antibiotic therapy judged necessary, or infection cleared with a negative culture result reported at the end of therapy); improved (partial resolution of clinical signs and symptoms or additional antibiotic therapy was necessary at the end of therapy not related to a worsening infection); failed (any of the following: inadequate response to therapy; resistant pathogen, worsening, or new or recurrent signs and symptoms; need for a change in antibiotic therapy; or a positive culture result reported at the end of therapy); or non-evaluable (unable to determine response at the end of therapy because the record did not contain adequate information). Follow-up visits were left to the discretion of the treating physician and patient compliance. Data was assessed using descriptive statistics. A Kaplan Meier analysis was used to assess time to clinical failure and survival estimates with the lower and upper 95% confidence intervals are presented. Estimation of 95% confidence intervals for proportions was made by the exact binomial method.

The safety of daptomycin was determined on the basis of the patient’s adverse events as reported by the investigator. All patients data were reviewed for adverse events during daptomycin and up to 30 days after completing therapy. The causal relationship between daptomycin treatment and the adverse event was described by the investigator as either not related (an adverse event with a temporal relationship to the drug administered that makes a causal relationship improbable, and/or for which other drugs or underlying or concurrent disease provide a plausible explanation) or possibly-related (a plausible temporal relationship to the drug administered, but for which other causative factor(s) could account for the event and where improvements on dechallenge or dose reduction may or may not have been observed).

Evaluable patients were included in the efficacy analysis. All patients were included in the safety analysis.

## Results

There were 209 patients with non-hardware-associated osteomyelitis that successfully completed daptomycin therapy during the study period. Seventy-three patients (36%) had follow-up information available and 71 patients had an evaluable clinical outcome. When data on follow-up assessments were collected, at least one year had passed since completion of daptomycin therapy. During that period of time, the median time of assessment was 35 days (min 1, max 798); 15 patients had assessments at ≥ 6 months (all resolved) and 5 had assessments at ≥ 1 year (all resolved). The failures occurred at 5, 38, 62 and 147 days after daptomycin therapy. Two non-evaluable patients were excluded from the efficacy analysis but were included in the safety analysis.

The patient demographics are shown in Table 1. Overall, the median daptomycin dose (min, max) was 6 mg/kg (4, 10) and the median duration of therapy was 42 days (1, 88). Twenty-four (34%) patients received less than 6 mg/kg and 47 (66%) patients received 6 or more mg/kg. The diagnosis of osteomyelitis was established or confirmed by the presence of the following: clinical signs and symptoms (n = 65), MRI (n = 40), bone scan (n = 7), plain radiography (n = 6), CT scan (n = 3), and cultures (primarily bone cultures; n = 29). The duration of osteomyelitis was not collected. Forty-five patients (63%) underwent a surgical intervention; including debridement (bone and tissue 16/71; 23% and tissue only 8/71; 11%). An additional 16 patients (23%) underwent incision and drainage.

**Table 1:** Patient Demographics in Evaluable Population

**Characteristics**	**Total (n = 71)**
Gender	
Female	36 (51)
Male	35 (49)
Age group (years)	
≤ 50	20 (28)
51-65	24 (33)
≥ 66	27 (38)
Weight, kg (median [min, max])	82 [37, 136]
Type of osteomyelitis	
Contiguous	45 (63)
Hematogenous	12 (17)
Unknown	14 (20)
Chronic presentation	29 (41)
Anatomic site	
Foot/Toe	38 (54)
Vertebrae	13 (18)
Sacrum	5 (7)
Sternum	4 (6)
Tibia/Fibula	3 (4)
Knee	2 (3)
Skull	2 (3)
Femur	1 (1)
Finger	1 (1)
Rib	1 (1)
Other	1 (1)
Multiple osteomyelitis sites	6 (8)
Concomitant infections	
Skin and skin structure infection†	9 (13)
Bacteremia	5 (7)
Deep surgical site infection	5 (7)
Urinary tract infection/pyelonephritis	3 (4)
Septic arthritis	1 (1)
Underlying diseases^*^	
Hypertension	34 (48)
Diabetes mellitus	31 (44)
Other cardiovascular disease	10 (14)
Peripheral vascular disease	13 (18)
Anemia/All hematologic diseases	10 (14)
Cardiac Arrhythmias	10 (14)
ICU stay during daptomycin	6 (9)
Initial CrCl < 30 ml/min	3 (4)
On dialysis	0 (0)

Data expressed as n (%) unless otherwise indicated.

† Types: major abscess n = 4, ulcer n = 2, wound n = 2, uncomplicated n = 1.

^*^ Patients may have more than one underlying disease.

Seventy Gram-positive pathogens were isolated in 52 patients (73%), primarily from deep tissue and bone cultures. *Staphylococcus aureus* was the most common pathogen (30/71; 42%), primarily MRSA (19/30; 63%). The other pathogens were coagulase-negative staphylococci (13/71; 18%), *Enterococcus* spp. (7/71; 10%), and *Streptococcus* spp. (3/71; 4%). Two patients had a Gram-negative or unidentified pathogen and the remainder reported negative cultures (n = 11) or no pathogen results (n = 6).

The use of concomitant antibiotics was documented in 43/71 patients (61%), primarily for Gram-negative and/or anaerobic coverage. The most common concomitant antibiotics were cephalosporins (15/43, 35%), carbapenems (11/43, 26%), and fluoroquinolones (11/43, 26%). Antibiotics were used after daptomycin in 31 patients (44%). Antimicrobials most commonly used included doxycycline (n = 8), linezolid (n = 6), and trimethoprim-sulfamethoxazole (n = 6). The median (min, max) duration of follow-up antibiotics was 29 days (4, 180).

**Figure 1:**
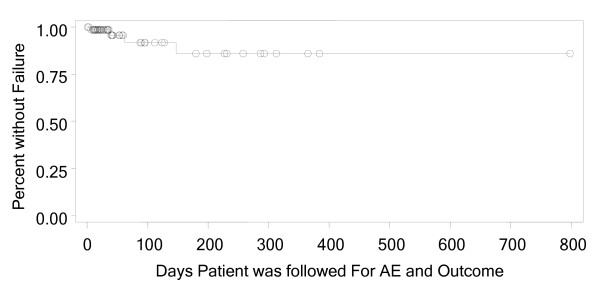
Kaplan Meier Analysis, Time to Clinical Failure Stratified by Dose of < 6 mg/kg and ≥ 6 mg/kg.

**Figure 2:**
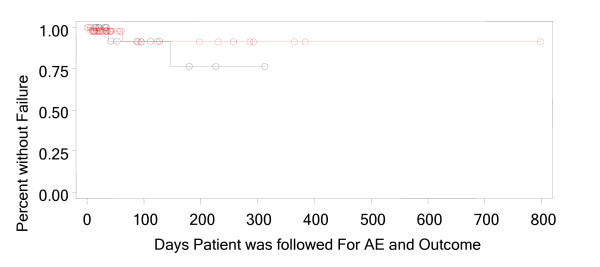
Kaplan Meier Analysis, Time to Clinical Failure.

Clinical resolution (resolved or improved) was reported in 94% (CI - 86.2%, 98.44%) of patients. The criteria used for outcome assessment included signs and symptoms (n = 67); laboratory tests such as white blood cell count, erythrocyte sedimentation rate (ESR) and C-reactive protein (CRP) (n = 19); radiologic tests (n = 12); and blood (n = 6) or other cultures (n = 3). A Kaplan Meier analysis was used to analyze time to clinical failure both overall and stratified by dose (Figure 1 and 2). The analysis of time to clinical failure showed that approximately 85% (CI – 64%, 95%) of patients had a continued successful outcome at the time of re-evaluation. The analysis of time to clinical failure showed that approximately 76% (CI – 31%, 94%) for less than 6 mg/kg and 91% (CI – 65%, 98%) for greater than equal to 6 mg/kg.

A subgroup analysis of 63 patients with confirmed osteomyelitis (radiologic evidence and/or bone culture), 52 patients with Gram-positive pathogens and 28 patients receiving daptomycin monotherapy showed success rates of 94% (CI - 85%, 98%), 96% (CI - 87%, 99%), and 96% (CI - 82%, 99%), respectively. The 18 patients meeting all 3 of these characteristics had a success rate similar to the entire cohort, 94% (CI - 73%, 99%). Additional details are provided as these patients outcomes may be more directly related to treatment with daptomycin. A review of their characteristics only found a higher rate of chronic renal failure (n = 4, 22%) in this group. Nine patients (50%) in this group had switched to daptomycin due to a clinical failure of prior therapy which was numerically higher than in the remainder of the cohort (12/53, 23%). There were 6 (33%) patients who received antibiotic therapy after daptomycin: cefazolin, trimethoprim/sulfamethoxazole, doxycycline (n = 3) and vancomycin. The one failure in this group occurred in a patient who received doxycycline for suppressive therapy of MRSA after daptomycin 6 mg/kg was administered for 70 days.

Thirty-six adverse events were reported in 18 (25%) patients. Seventeen adverse events possibly related to daptomycin occurred in 13 (18%) patients (Table 2). Creatine phosphokinase (CPK) values were determined at baseline in 29 (40%) patients and during daptomycin therapy in 63 (89%) patients, primarily weekly. One patient each had a peak CPK value >5 to 10 times and >10 times the upper limit of normal, both resolved while on daptomycin therapy. Three (4%) patients discontinued daptomycin treatment as a result of an adverse event. All adverse events were mild to moderate in intensity.

**Table 2:** Possibly-Related Adverse Events in the Safety Population

**Adverse Event**	**N (%)**	**Serious**	**Action Taken**	**Resolution**
Blood CPK Increased	8 (11)	No	None	Resolved
		No	None	Resolved
		No	None	Resolved
		No	None	Resolved
		No	None	Resolved
		No	None	Resolved
		No	None	Unknown
		Yes	Dose Reduced	Resolved
Diarrhea	3 (4)	No	None	Unknown
			None	Resolved
			Stopped Permanently	Resolved
Rash	2 (3)	No	Stopped Permanently	Resolved
Chills	1 (1)	No	Stopped Permanently	Resolved
Nausea	1 (1)	No	None	Resolved
Photosensitivity Reaction	1 (1)	No	Stopped Temporarily	Resolved
Pyrexia	1 (1)	No	Stopped Permanently	Resolved

Subjects may have had more than one possibly related AE.

All adverse events were reported by the investigator.

## Discussion

There is limited data on the outcomes of patients with bone and joint infections that were treated with daptomycin. Recent data indicates that vancomycin activity against MRSA has decreased [4]. The current study of patients with osteomyelitis was designed to collect outcome data on patients treated with daptomycin, an alternative to vancomycin.

There are several limitations inherent to the retrospective nature of this study, including the potential for patient selection bias. However, the demographics of these patients are similar to previously published studies with predominantly older patients, with almost 50% with diabetes mellitus and peripheral vascular disease [6]. Although the study was conducted at a time when all previously treated patients would have completed at least one year of follow-up assessment, approximately two-thirds of the daptomycin treated patients could not be included in this analysis due to a lack of an outcome assessment. It is difficult to assess how a higher rate of follow-up would have influenced the results. This study collected outcomes based on the standard of care practiced at each institution, as such; diagnostic procedures, daptomycin dose and duration, and surgical interventions were uncontrolled.

Clinical resolution was documented at the follow-up assessment in 94% of patients in this study. Further, approximately 85% of patients in a Kaplan Meier analysis did not have recurrence of their infection. However, our data should be considered in the context of the small sample size and the amount of censored data in the study. These results are similar to previous studies. Finney et al. evaluated daptomycin in a non-comparative study of early clinical experience with bone and joint infections. Seven osteomyelitis patients were treated with daptomycin at doses ranging from 4 to 6 mg/kg, for a duration of therapy between 8 to 42 days. Six of 7 patients had MRSA. The duration of follow-up was not reported. All patients had a successful resolution of signs and symptoms [15]. Antony and colleagues reported on their clinical experience with daptomycin for the treatment of patients with Gram-positive orthopedic infections. Of the 31 patients treated, 16 were diagnosed with osteomyelitis; 14 of those had MRSA. Most patients (77%) were treated with daptomycin at a dose of 6 mg/kg; the remainder received 4 mg/kg. The overall cure rate was 87% and 100% for the 16 patients with osteomyelitis after 4 to 6 months of follow-up [16]. Shipton et al. reported a case series of 7 patients with osteomyelitis treated with daptomycin at a dose of 4 or 6 mg/kg for a duration between 11 days to 8 weeks. Treatment was successful in 3 (43%) cases after completing daptomycin and follow-up of a mean of 7.3 months (range, 6–9 months). Daptomycin in vitro non-susceptibility was reported in 1 patient [17]. A recent retrospective study by Licitra et al. investigated the outcome of osteomyelitis or prosthetic joint infections (PJI) patients after treatment with daptomycin at a dose of ≥6 mg/kg for at least 2 weeks, (median 49 days; range, 21–183). All patients had a Gram-positive pathogen. Fifty-five of 59 (93%) osteomyelitis patients and 14/14 (100%) PJI patients were clinically cured or improved at 6 months after completing daptomycin [18].

This current study did not find that the outcomes of patients with osteomyelitis and treated with daptomycin was affected by the dose of <6 mg/kg compared to ≥6 mg/kg. There is scant data in the literature to determine the optimal daptomycin dose in osteomyelitis. An earlier study of a different cohort in the CORE registry of osteomyelitis patients reported a successful outcome in 82% of patients after a median follow-up of 9 weeks. Clinical success was more common at a daptomycin dose >4 mg/kg than at dosages ≤4 mg/kg (88% vs. 65%; p = 0.013) [12]. Rao et al. reported that daptomycin administered at a dose of 4 mg/kg in 12 patients with PJI had a success rate of 50% (defined as no clinical or radiographic recurrence; continued decline in ESR and CRP levels; and continued improvement of joint function) after follow-up of 8 to 13 months. Most of the reported failures retained hardware. This fact and the lower dose of daptomycin used in this cohort may have contributed to the low success rate [19]. As demonstrated, several studies have shown a favourable clinical outcome and safety profile in patients with osteomyelitis and PJI that were treated with daptomycin [12,15,16,20,21]; however, failures including the development of resistance have been reported [19,22,23].

The safety profile of daptomycin in this study compares favorably to previous reports. The discontinuation rate of 4% is similar to that reported in several studies of osteomyelitis; 4% (3/73) by Licitra et al., 3% (1/31) by Antony et al., 0% (0/12) by Rao et al., and 0% (0/36) by Hernandez et al. [16,18,19,24]. The rate of CPK elevations was 11% (8/73); however, none discontinued and only one patient had their daptomycin dose reduced. This rate is somewhat higher than reported in the literature, which has ranged from 0% in smaller case series to 8% [16,18,19,24]. This variability may have been the result of several factors. Not all investigations have used the same frequency of testing for CPK, ranging from weekly examinations as recommended in the daptomycin prescribing information to no routine testing of CPK values. Furthermore, the cut-off values of significant CPK elevation were variable.

## Conclusions

In conclusion, daptomycin appears to be an effective therapeutic choice for patients with osteomyelitis and has an acceptable safety profile. Further studies looking at the optimal dosage of daptomycin for patients with osteomyelitis are warranted.

## Competing interests

One or more of the authors has received funding from Cubist Pharmaceuticals. JAH, DC, SAM, and MY are all employees and stock holders at Cubist Pharmaceuticals.

## Authors’ contributions

JCG: participated in its design and coordination and helped to review the manuscript. JAH: participated in its design and coordination and helped to draft and review the manuscript. DC: participated in its design and coordination and helped to review the manuscript. SAM: participated in its design and coordination and helped to review the manuscript. MY: participated in the design of the study and performed the statistical analysis. EB: participated in its design and coordination and helped to review the manuscript. All authors read and approved the final manuscript.

## Pre-publication history

The pre-publication history for this paper can be accessed here:

http://www.biomedcentral.com/1471-2334/12/133/prepub
